# Thionation-enhanced through-space electronic coupling and excited-state dynamics in helicene diimides

**DOI:** 10.1039/d6cc01559j

**Published:** 2026-04-27

**Authors:** Analia D’Orazio-Colman, Amalnadh T., Sona Saji, Mahesh Hariharan, Prince Ravat

**Affiliations:** a Julius-Maximilians-Universität Würzburg, Institut für Organische Chemie Am Hubland D-97074 Würzburg Germany; b School of Chemistry, Indian Institute of Science Education and Research Thiruvananthapuram (IISER TVM) Thiruvananthapuram 695551 Kerala India; c Department of Chemistry and Biochemistry, Institute of Organic Chemistry, University of Cologne 50939 Cologne Germany pravat@uni-koeln.de mahesh@iisertvm.ac.in

## Abstract

Thionation provides a simple heteroatom substitution strategy to enhance through-space electronic coupling in helically constrained π-systems. A thionated [8]helicene diimide shows pronounced LUMO stabilization and nearly doubled electronic coupling between the spatially aligned imide units. Spectroscopic and computational studies reveal enhanced charge delocalization and altered excited-state dynamics.

## Abstract 2

Thionation strengthens through-space coupling in helicene diimides, boosting intervalence charge transfer and reshaping excited-state relaxation pathways.

## Graphical abstract

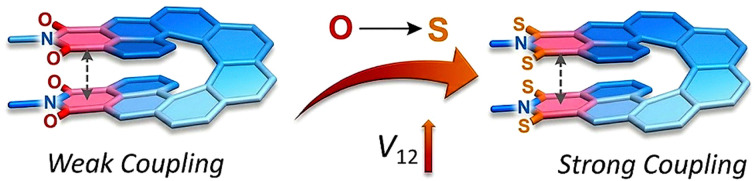

Rylene diimides are prototypical π-conjugated electron acceptors widely used in organic electronics owing to their chemical robustness, high electron affinity, and well-defined redox behavior.^1^ Their rigid π-frameworks enable efficient electronic communication between redox centres, making them attractive platforms for studying charge transport, mixed-valence states, and intervalence charge transfer (IVCT), as well as for applications in n-type semiconductors and optoelectronic devices.^2^ Thionation of imide carbonyl groups provides an effective strategy to tune their electronic structure without modifying the conjugated backbone.^3^ In naphthalene and perylene diimides, sulfur substitution induces LUMO stabilization, enhanced polarizability, and bathochromic shifts arising from the diffuse 3p orbitals of sulfur, which can strengthen electronic coupling and charge delocalization.^4^

Beyond planar architectures, helicene-based π-systems introduce additional electronic interactions arising from their nonplanar, helical topology and intrinsic chirality.^5^ Incorporation of imide functionalities into helicenes has produced helicene diimides ([*n*]HDIs),^6^ which combine electron-deficient character with rigid chiral frameworks.^7^ In these systems, electronic communication between terminal imide units arises from pronounced intramolecular through-space interactions. Our recent work showed that this coupling strongly depends on the helical pitch, with [8]helicene diimide ([8]HDI_O4) representing a limiting case where the imide units are brought into close spatial proximity, enabling enhanced through-space conjugation and IVCT.^6*c*^ Despite this favourable geometry, strategies to further amplify through-space electronic interactions in helically constrained π-systems remain limited.^8^

Here we explore whether heteroatom substitution can provide an additional handle to strengthen these interactions.^9^ We report the synthesis of a thionated [8]helicene diimide ([8]HDI_S4) and compare it with the oxo analogue ([8]HDI_O4). Spectroelectrochemical and ultrafast spectroscopic studies reveal that thionation significantly enhances through-space electronic coupling, promotes charge delocalization, and modifies excited-state dynamics, establishing thionation as an effective strategy to modulate through-space charge-transfer processes in helically constrained π-systems and advancing chiral n-type organic materials.

The helicene diimide precursor^6*c*^ [8]HDI_O4 was converted to the thionated analogue [8]HDI_S4 through selective tetrathionation of the four imide carbonyl groups (Scheme 1). Reaction conditions were carefully optimized to avoid partial thionation, which otherwise produces mixtures of regioisomers. Treatment of *rac*-[8]HDI_O4 with eight equivalents of recrystallized Lawesson's reagent in refluxing toluene for 72 h afforded *rac*-[8]HDI_S4 in 67% yield.^10^ Enantiopure samples of [8]HDI_S4 were subsequently obtained from the corresponding enantiopure [8]HDI_O4 precursor with retention of configuration owing to the high configuration stability of [8]helicene backbone.^5*c*,11^

**Scheme 1:**
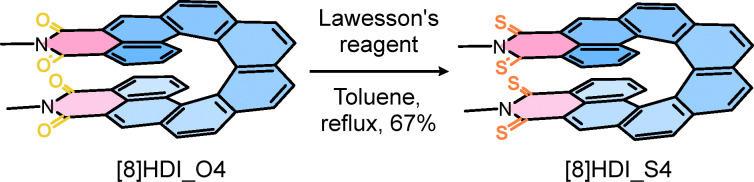
Synthesis of [8]HDI_S4.

The structural differences between [8]HDI_O4 and [8]HDI_S4 were analysed using DFT-optimized geometries (ωB97XD/6-31g(d,p)). The C=S bond in [8]HDI_S4 (1.66 Å) is significantly longer than the C=O bond in [8]HDI_O4 (1.22 Å), consistent with typical thiocarbonyl and carbonyl bond lengths. This reflects the larger atomic radius and more diffuse 3p orbitals of sulfur relative to the 2p orbitals of oxygen, resulting in reduced π overlap. At the same time, the greater polarizability and spatial extension of sulfur orbitals enhance electronic delocalization within the π-framework, stabilizing the LUMO and increasing charge-transfer character (Fig. 2c).^12^ In [8]HDI, this orbital diffuseness is expected to strengthen through-space electronic coupling between the imide units, promoting charge delocalization and intervalence charge transfer.^6*a*,8*a*,8*c*,8*d*^

Whereas the reference compound [8]HDI_O4 is obtained as a yellow solid, the thionated derivative [8]HDI_S4 appears as a black powder, reflecting pronounced changes in optical properties upon sulfur substitution. In solution, [8]HDI_O4 is yellow, while [8]HDI_S4 displays an orange-red colour, consistent with a substantial red shift in absorption. UV-vis spectra recorded in DCM show a pronounced bathochromic shift upon thionation, accompanied by a reduction of the optical energy gap from 2.53 eV to 1.77 eV (Fig. 1). Both compounds exhibit only minor changes in absorption onset with solvent polarity (SI Fig. S4), indicating negligible ground-state charge-transfer character. This red shift parallels trends reported for thionated rylene diimides, where sulfur substitution similarly reduces the band gap.

**Fig. 1:**
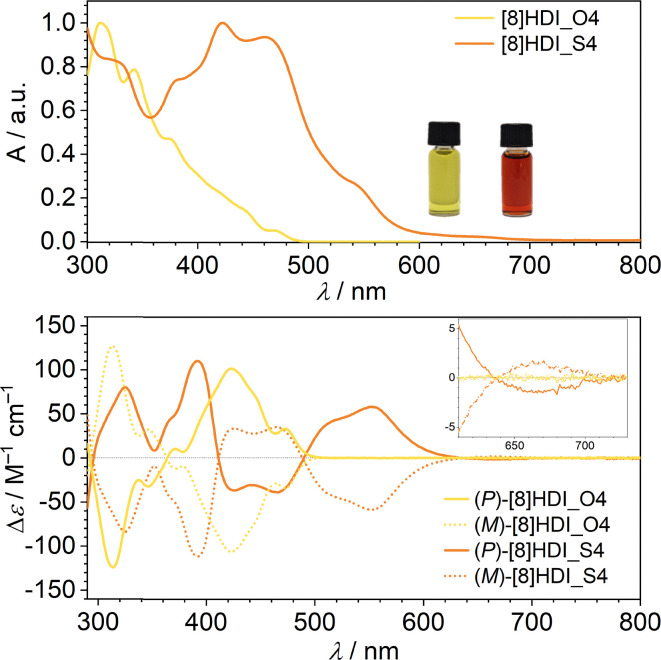
Normalized absorption spectra (top) and ECD (bottom) of [8]HDI_O4 and [8]HDI_S4 in DCM (*c ∼* 10^−5^ M).

These spectral features are reproduced by TD-DFT calculations (B3LYP/6-311G(2d,p)), which reveal clear differences in the low-energy excited states. For [8]HDI_S4, the lowest singlet states are strongly red-shifted with low oscillator strengths due to multiconfigurational mixing of frontier orbitals (HOMO−1 → LUMO, HOMO−2 → LUMO, HOMO → LUMO+1), consistent with enhanced charge-transfer character and electronic delocalization (SI Table S4). In contrast, [8]HDI_O4 shows higher-energy transitions with larger oscillator strengths characteristic of localized π–π* excitations. Thionation also reorganizes the frontier orbitals: whereas those of [8]HDI_O4 are mainly delocalized over the helicene backbone with minimal oxygen contribution, the C=S groups introduce significant sulfur participation with low-lying nonbonding character. The greater spatial extension of these orbitals enhances orbital mixing and compresses the Frontier orbital manifold, thereby increasing electronic coupling (SI Fig. S12).^4*b*,13^

The chiroptical properties of [8]HDI_O4 and [8]HDI_S4 were investigated by circular dichroism (CD) spectroscopy in DCM. Absolute configurations were assigned by comparison of the experimental spectra with TD-DFT calculations (Fig. 1, SI Fig. S13–S14). Both compounds exhibit absorption dissymmetry factors (*g*_abs_) on the order of 10^−2^ at the lowest-energy transitions, and the enantiomers display perfectly mirrored CD spectra with opposite Cotton effects. Notably, [8]HDI_S4 shows a red-shifted and more intense CD signal in the 500–600 nm region compared to [8]HDI_O4, consistent with enhanced electronic delocalization upon thionation.

To evaluate the impact of thionation on electrochemical properties and frontier orbital energies, cyclic voltammetry (CV) and differential pulse voltammetry (DPV) were performed in DCM (Fig. 2). Both compounds exhibit two reversible reduction waves corresponding to the sequential reduction of the two imide units. Thionation significantly stabilizes the LUMO, shifting from −3.45 eV for [8]HDI_O4 to −3.97 eV for [8]HDI_S4, while the separation between the reduction potentials remains unchanged (Δ*E*_red_ = 0.22 V). This large separation corresponds to a comproportionation constant (*K*_c_) of 5.1 × 10^3^ indicating substantial thermodynamic stabilization of the mixed-valence radical anion with respect to disproportionation.^14^

**Fig. 2:**
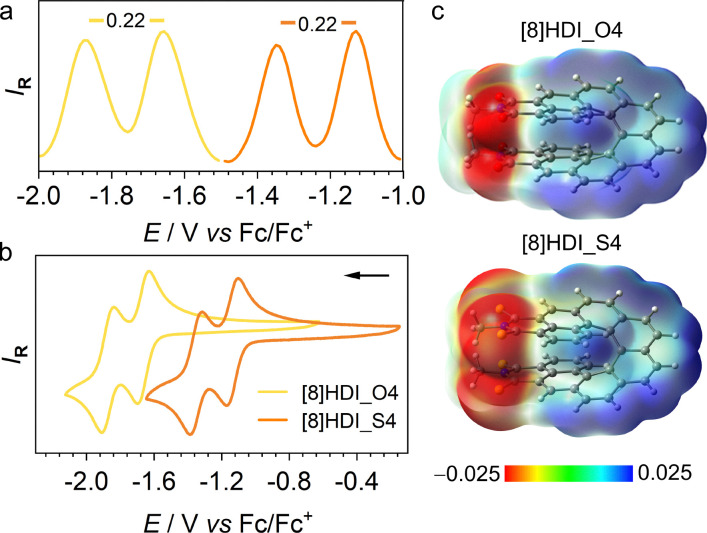
(a) DPV and (b) CV plots *vs.* Fc/Fc^+^ in DCM with a supporting electrolyte, [Bu_4_N][PF_6_] (0.2 M), at a scan rate of 200 mV s^−1^ for CV and 25 mV s^−1^ for DPV. (c) Molecular electrostatic potential (MEP) map.

To directly probe changes in electronic coupling between the imide units, spectroelectrochemical (SEC) measurements were performed (SI Fig. S7). Both compounds exhibit broad near-infrared (NIR) intervalence charge-transfer (IVCT) bands (1500–3000 nm) in their mixed-valence states; however, this band is slightly more intense and shifted to higher energy for [8]HDI_S4, indicating stronger electronic interaction (Fig. 3). Quantitative analysis of the IVCT bands reveals that the electronic coupling (*V*_12_) increases from 1346 cm^−1^ for [8]HDI_O4 to 2921 cm^−1^ for [8]HDI_S4, corresponding to an approximately twofold enhancement upon thionation. The reorganization energy for [8]HDI_S4 (*λ* ∼ 4668 cm^−1^) satisfies the condition *V*_12_ > *λ*/2, placing the system near the adiabatic limit and consistent with movement toward the Class II/III boundary with substantial charge delocalization.^15^ In contrast, [8]HDI_O4 (*λ* ∼ 4493 cm^−1^, *V*_12_ < *λ*/2) remains in the nonadiabatic regime, consistent with a more localized Class II system.

**Fig. 3:**
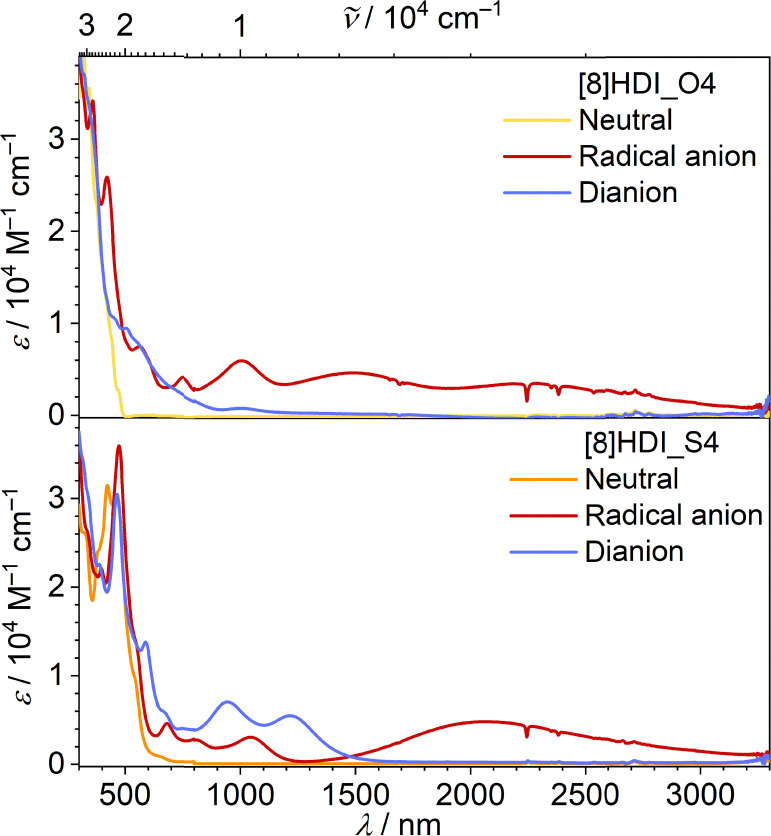
Fitted spectral components from SEC measurements (SI Fig. S7) corresponding to the neutral, radical anion, and dianion.

DFT calculations of the IVCT parameters for the radical anion were performed using a hybrid functional containing 35% exact Hartree–Fock exchange.^16^ The computed results reproduce the experimental trends, predicting stronger electronic coupling for [8]HDI_S4 together with a significantly lower activation free energy for electron transfer (SI Table S5). These results indicate faster electron transfer in the thionated derivative. Overall, the combined experimental and computational data demonstrate that thionation enhances electronic coupling and charge delocalization in helicene diimides.

Femtosecond transient absorption (fs-TA) spectroscopy was used to probe the excited-state dynamics of [8]HDI_O4 and [8]HDI_S4 in toluene (TOL) and chloroform (CHL) (Fig. 4). For [8]HDI_O4 in TOL, 400 nm excitation with a 100-fs pump pulse generated transient features over 435–720 nm. Global analysis with a sequential A → B → C → GS kinetic model resolved three species: solvent relaxation after charge-transfer (CT) excitation (∼19 ps), a hot singlet CT state (∼256 ps), and a relaxed singlet CT state persisting beyond the experimental window (>2.9 ns). This long-lived state is consistent with the fluorescence lifetime in TOL (*τ*_f_ ≈ 5.72 ns).^6*c*^ In CHL, only two components were identified: an excited singlet CT state (∼251 ps) and a long-lived relaxed singlet CT state (>2.9 ns).^6*b*^ The initially populated transient species of [8]HDI_O4 in TOL is absent in CHL, likely due to the faster solvent relaxation dynamics in the more polar CHL (SI Fig. S8a). This rapid relaxation stabilizes the charge-transfer state at earlier timescales, thereby preventing the deconvolution of the initial relaxation process.^17^

**Fig. 4:**
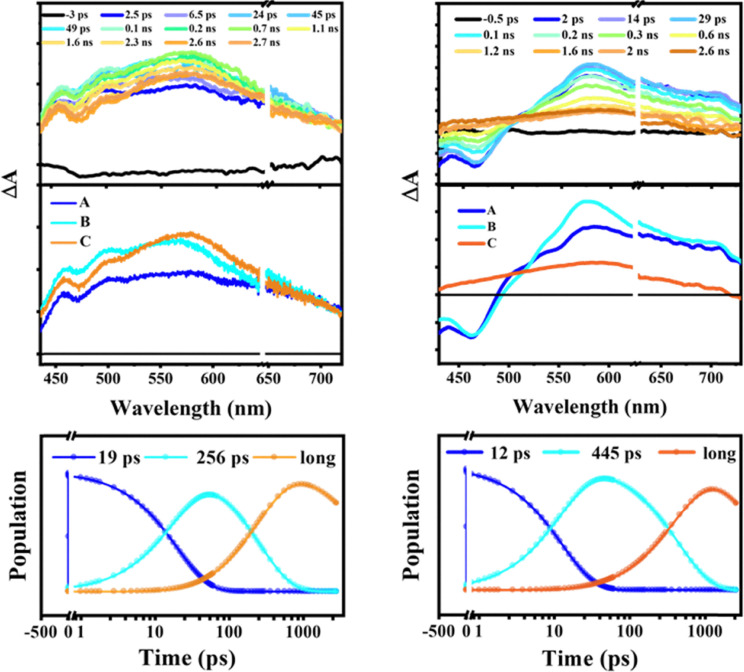
(Top row) fs-TA spectra showing the excited state dynamics upon photoexcitation (*λ*_ex_ = 400 nm) in toluene. (Middle row) evolution associated spectra (EAS) reconstructed from global analysis with A → B → C → GS model. (Bottom row) relative population profiles of the excited states. [8]HDI_O4 (left) and [8]HDI_S4 (right).

For [8]HDI_S4, fs-TA measurements likewise showed solvent-dependent dynamics. In TOL, three components were resolved: solvent relaxation (∼12 ps), a singlet CT state (∼445 ps), and a spectrally distinct long-lived species beyond 2.9 ns, likely a triplet state. In CHL, only two components were observed: a singlet CT state decaying within ∼240 ps and a long-lived species persisting beyond the time window. The absence of a resolved solvent-relaxation component and the shorter S_1_ lifetime in CHL indicate faster singlet-state depopulation (SI Fig. S8b). Overall, the faster generation of the long-lived species in [8]HDI_S4 supports thionation-enhanced electronic coupling and stronger CT character, consistent with the IVCT analysis.^18^

Nanosecond transient absorption (ns-TA) spectroscopy was used to probe the fate of the CT singlet state of [8]HDI_O4 in TOL and CHL (SI Fig. S9). Upon 355 nm excitation with an 8–10 ns pulse, nitrogen-purged solutions showed a broad excited-state absorption (ESA) band centred at ∼600 nm, assigned to the helicene diimide triplet state. Its rapid quenching under oxygen confirmed the triplet nature. Monoexponential fitting of the decay at 600 nm gave triplet lifetimes of 7.37 µs in TOL and 18.90 µs in CHL (SI Fig. S10).

To clarify the nature of the long-lived species in [8]HDI_S4 observed by fs-TA, ns-TA measurements were performed in nitrogen-purged TOL and CHL (SI Fig. S11). Upon 355 nm excitation, the spectra showed mainly ground-state bleach signals without detectable positive ESA, suggesting a very short-lived or weakly populated triplet state whose absorption is likely masked by the dominant ground-state bleach signal.^19^ This behavior indicates that thionation strongly modifies the excited-state relaxation pathway, consistent with previous reports on thionated chromophores.^20^ Supporting this, spin–orbit coupling (SOC) calculations revealed weak SOC for [8]HDI_O4 (∼0.18 cm^−1^) but much larger SOC for [8]HDI_S4 (∼48.50 cm^−1^), consistent with more efficient intersystem crossing promoted by sulfur (Tables S10 and S11).^21^ Hence, thionation not only enhances charge transfer but also alters triplet-state dynamics through increased SOC.

In summary, using [8]HDI—where the imide units are brought into close spatial proximity—we probed the influence of heteroatom substitution on through-space charge-transfer processes within a three-dimensional framework. Spectroelectrochemical analysis reveals a pronounced increase in electronic coupling (*V*_12_) upon thionation, moving the system toward the Robin–Day Class II/III boundary and indicating enhanced charge delocalization between the imide units. Ultrafast transient absorption measurements further reveal accelerated charge-transfer formation and altered excited-state relaxation pathways in the thionated [8]HDI derivative compared to the non-thionated analogue. While [8]HDI_O4 exhibits a long-lived triplet state, the thionated analogue undergoes faster singlet-state depopulation due to enhanced electronic communication and increased spin–orbit coupling, resulting in the absence of long-lived triplet signatures. Broadly, these results demonstrate that thionation provides an effective strategy to control through-space electronic interactions and excited-state dynamics in helically constrained architectures, offering design guidelines for chiral n-type organic semiconductors with tuneable charge-transfer properties.

## Conflicts of interest

There are no conflicts to declare.

## Supplementary Material

CC-062-D6CC01559J-s001

## Data Availability

All experimental procedures, characterization data, spectroscopic analyses, and computational details are provided in the supplementary information (SI). Supplementary information is available. See DOI: https://doi.org/10.1039/d6cc01559j.
