# Supplementary material for: Thionation-enhanced through-space electronic coupling and excited-state dynamics in helicene diimides
Source: Chem Commun (Camb). 2026 Apr 27;62(40):10216–20. doi: 10.1039/d6cc01559j (PMC13147499; doi:10.1039/d6cc01559j)
Supplement: CC-062-D6CC01559J-s001 [file CC-062-D6CC01559J-s001.pdf]

Supplementary Information (SI) for ChemComm.
This journal is © The Royal Society of Chemistry 2026

Supporting information for

Thionation-Enhanced Through–Space Electronic Coupling and Excited–State Dynamics in Helicene Diimides

Analia D'Orazio-Colman,[a] Amalnadh T,[b] Sona Saji,[b] Mahesh Hariharan,[b*] and Prince Ravat[c*]

[a]Julius-Maximilians-Universität Würzburg, Institut für Organische Chemie, Am Hubland, D-97074 Würzburg, Germany.

[b]School of Chemistry, Indian Institute of Science Education and Research Thiruvananthapuram (IISER TVM), Thiruvananthapuram 695551 Kerala, India

[c]Department of Chemistry and Biochemistry, Institute of Organic Chemistry, University of Cologne, 50939 Cologne, Germany

Email: pravat@uni-koeln.de; mahesh@iisertvm.ac.in

Author contribution

A.D.C. performed the synthesis, characterization, UV–vis, and (spectro)electrochemical measurements, and prepared the first draft of the manuscript and ESI. A.T. performed the fs-TA and ns-TA measurements and analysed the data. S.S. carried out the SOC calculations. M.H. supervised the TA experiments and contributed to writing the transient absorption and excited state analysis sections of the manuscript. P.R. conceived the project, supervised and coordinated the research, performed (TD)-DFT calculations, and prepared the final version of the manuscript. All authors discussed the results and commented on the manuscript.

Table of contents

S1. Experimental Details 2
S2. Synthesis 5
S3. Spectroscopy 7
S4. Electrochemistry 9
S5. Spectroelectrochemistry 10
S6. Transient Absorption 12
S7. Quantum Chemical Calculations 14
S8. References 21

S1. Experimental Details

Synthesis and Materials:

All reagents and solvents were obtained from commercial suppliers and used as received, unless otherwise specified. Racemic and enantiopure, [8]HDI_O4 were synthesized according to literature known protocols.[1] Reactions and experiments sensitive to oxygen were carried out under a nitrogen atmosphere using Schlenk techniques and solvents saturated with nitrogen.

Chromatography:

Open-column chromatography and thin-layer chromatography (TLC) were performed on silica gel (Merck silica gel 60M, 40–63 µm).

NMR Spectroscopy:

The NMR experiments were performed at 298 K on NMR spectrometers operating at 400 MHz ^{1}H and 101 MHz ^{13}C frequencies. Standard pulse sequences were used, and the data was processed using 2-fold zero-filling in the indirect dimension for all 2D experiments. Chemical shifts (δ) are reported in parts per million (ppm) relative to the solvent residual peak (^{1}H and ^{13}C NMR, respectively): $CDCl_3$ (δ = 7.26 and 77.16 ppm), and J values are given in Hz.

HRMS:

MALDI-TOF-HRMS were measured on a Bruker ultrafleXtreme mass spectrometer. *Trans*-2-[3-(4-*tert*-butylphenyl)-2-methyl-2-propenylidene]malononitrile (DCTB) dissolved in chloroform (30 mg/mL) was used as supporting matrix in the MALDI–TOF–HRMS measurement. Reference spectra were simulated using the mMass software.[2]

UV–vis Absorption Spectroscopy:

UV–vis spectra were measured on a JASCO V–670 spectrophotometer.

Electronic Circular Dichroism

Electronic Circular Dichroism (ECD) spectra were recorded on either a Jasco J-810 CD spectropolarimeter at 293 K.

Cyclic voltammetry and Differential Pulse Voltammetry:

Cyclic voltammetry and Differential Pulse Voltammetry (DPV) experiments were performed in DCM with 0.2 M $[Bu_4N][PF_6]$ as supporting electrolyte, using a Gamry Instruments Reference 600 potentiostat. A standard three-electrode cell configuration was employed, using a platinum disk working electrode, a platinum wire counter electrode, and a platinum wire

serving as reference electrode. The redox potentials were referenced to the ferrocene (Fc) / ferrocenium (Fc^+) redox couple.

Spectroelectrochemistry (SEC):

Spectroelectrochemical absorption measurements were carried out in reflection mode using an Agilent Cary 5000 UV/Vis/NIR spectrophotometer equipped with a custom-made sample compartment. The setup included a cylindrical PTFE cell with an optical window and an adjustable two-in-one electrode composed of a 6 mm platinum disc working electrode and a 1 mm platinum wire counter electrode, along with a leak-free Ag/AgCl reference electrode. The optical path length was set to 100 µm using a micrometer screw. Potentials were applied using a PalmSens EmStat4s potentiostat. All experiments were performed at room temperature under an argon atmosphere with 0.1 M $[Bu_4N][PF_6]$ as the supporting electrolyte.

Femtosecond Transient Absorption (fs-TA) Measurement:

Femtosecond transient absorption measurements of **[8]HDI_O4** and **[8]HDI_S4** in toluene and chloroform were carried out using a Mai Tai SP mode-locked laser operating at 86 MHz and emitting at 800 nm as the seed source for a Spitfire Ace regenerative amplifier (Spectra-Physics). The regenerative amplifier operated at a repetition rate of 1 kHz and delivered pulse energies of 5.5 mJ. A portion of the amplified 800 nm output was frequency-doubled in a Beta barium borate (BBO) crystal to generate 400 nm pump pulses via second-harmonic generation (SHG). The remaining 800 nm beam passed through an optical delay stage in an ExciPro pump–probe spectrometer. A sapphire crystal placed in this path produced a white-light continuum, which was subsequently divided into probe and reference beams. Transient absorption spectra were recorded using a dual diode-array detector with a 200 nm detection window and a maximum optical delay of 2.9 ns. Samples were contained in a rotating cuvette with a 1.2 mm path length. The instrument response function (IRF) was determined from the two-photon absorption signal of a solvent mixture (10% benzene in methanol) and was found to be approximately 110 fs at ~530 nm. An 80% neutral density filter was used to control the excitation fluence. For the fs-TA experiments, samples were excited at 400 nm with 100 fs pulses of 200 nJ energy. The kinetic components were independent of excitation intensity, ruling out singlet–singlet annihilation effects. All measurements were performed at the magic angle (~54.7°) between pump and probe polarizations.

Global Analysis

The fs-TA data of **[8]HDI_O4** and **[8]HDI_S4** in toluene and chloroform were analyzed

globally using the Glotaran software package. This analysis incorporated correction for the instrument response function (IRF) and group velocity dispersion (GVD) of the white-light continuum, allowing accurate extraction of decay time constants and dispersion-compensated spectra. In the global fitting procedure, kinetic traces at all wavelengths were analyzed simultaneously using a sequential model to obtain evolution-associated spectra (EAS). It should be noted that EAS represents the time-dependent spectral evolution of the system and do not necessarily correspond to distinct physical or chemical species. Rather, they reflect spectral changes characterized by their respective time constants.

Nanosecond Transient Absorption (ns-TA) Measurement

Nanosecond laser flash photolysis experiments were performed on nitrogen-purged solutions of **[8]HDI_O4** and **[8]HDI_S4** in toluene and chloroform. The measurements were conducted using an LKS-60 from Applied Photophysics. Excitation was provided by the third harmonic (355 nm, ~10 ns pulse width) of a Quanta Ray INDI-40-10 pulsed Nd:YAG laser. A break was introduced in the spectra at 532 nm, when necessary, to avoid interference from the residual second harmonic of the nanosecond laser pulse. The transient decay profiles obtained from the ns-TA measurements were monitored at 600 nm and analyzed by exponential fitting using OriginPro. The instrument response function (IRF) was determined to be approximately 10 ns.

S2. Synthesis of [8]HDI_S4

Under a nitrogen atmosphere, **[8]HDI_O4** (0.100 g, 0.168 mmol, 1 eq.) and Lawesson's reagent (0.544 g, 1.35 mmol, 8 eq.) were added to a Schlenk tube. The mixture was dissolved in 50 mL of dry toluene and stirred at 110 °C for 72 hours. After cooling to room temperature, the solvent was removed under reduced pressure. The crude residue was extracted with dichloromethane and washed several times with brine and water. The organic phase was dried over anhydrous sodium sulphate and filtered through a Celite plug. The solvent was then removed under reduced pressure, and the residue was purified by column chromatography using chloroform/acetone (10:0.5) as the eluent. The product was further purified by dissolving it in a minimal amount of chloroform followed by precipitation in methanol, yielding a black crystalline powder (75 mg, 0.114 mmol, 67%). The reaction was performed in the same manner for both racemic and enantiomerically pure starting materials, affording racemic and enantiopure **[8]HDI_S4**, respectively. The high inversion barrier associated with the enantiomerization of the [8]helicene framework ensures configurational stability under the applied reaction conditions. Since the thioimide units are located on the outer rim of the helicene framework, we expect thionation to have only a negligible effect on the configurational stability of the [8]helicene core, as the outer rim is not directly involved in the helix-inversion process.[3] Consequently, the target compound **[8]HDI_S4** is obtained with retention of the original enantiopurity.

^{1}H NMR (400 MHz, $CDCl_3$): δ [ppm] = 8.56 (dd, *J* = 7.7, 1.1 Hz, 1H), 8.49 (s, 1H), 8.13 (d, *J* = 8.3 Hz, 1H), 7.99 (d, *J* = 8.3 Hz, 1H), 7.83 (d, *J* = 8.3 Hz, 1H), 7.58 (d, *J* = 8.5 Hz, 1H), 7.36 (dd, *J* = 8.3, 1.1 Hz, 1H), 6.64 (dd, *J* = 8.3, 7.7 Hz, 1H), 4.52 (s, 3H).

^{13}C NMR (101 MHz, $CDCl_3$): δ [ppm] = 191.8, 190.9, 138.5, 135.3, 134.2, 131.8, 130.7, 130.6, 129.05, 129.0, 128.4, 128.1, 128.0, 127.7, 127.0, 125.9, 125.7, 124.1, 121.7, 44.2.

HRMS (MALDI, negative mode) m/z: $[M]^-$ calculated for $C_{40}H_{22}N_2S_4$ 658.0666; found 658.0690 (Δ = 3.65 ppm).

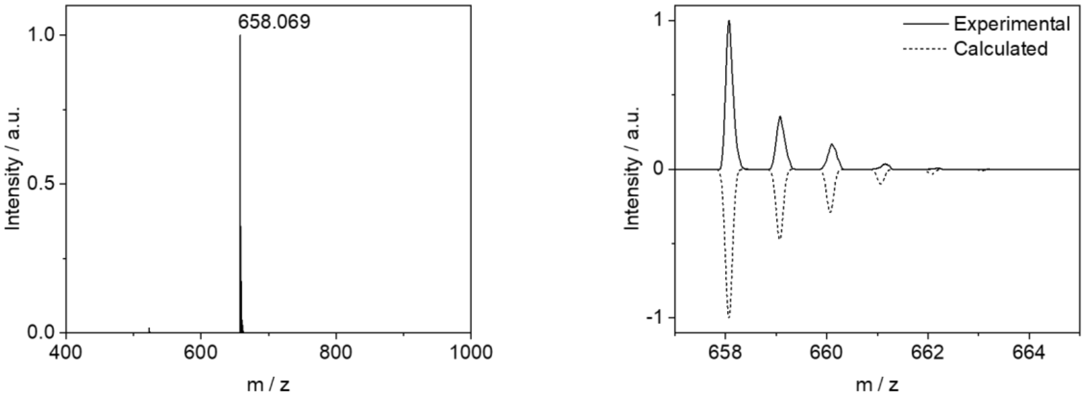

Figure S1. MALDI-TOF HRMS of **[8]HDI_S4**.

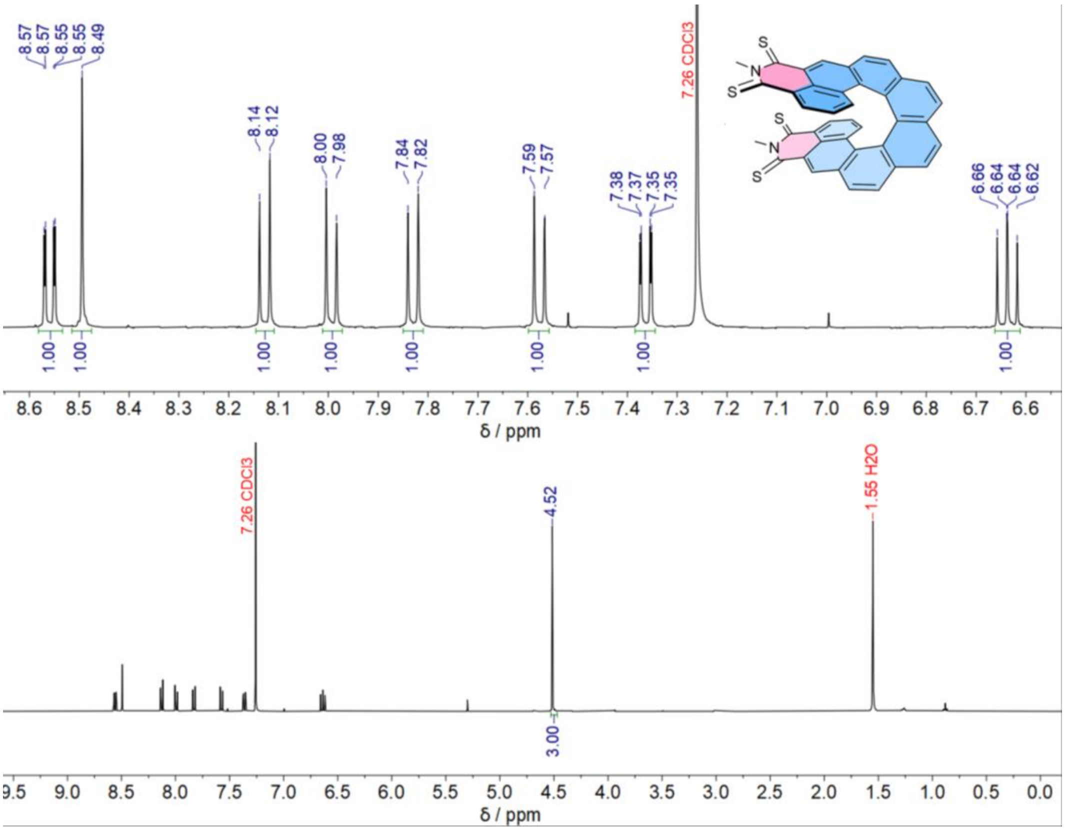

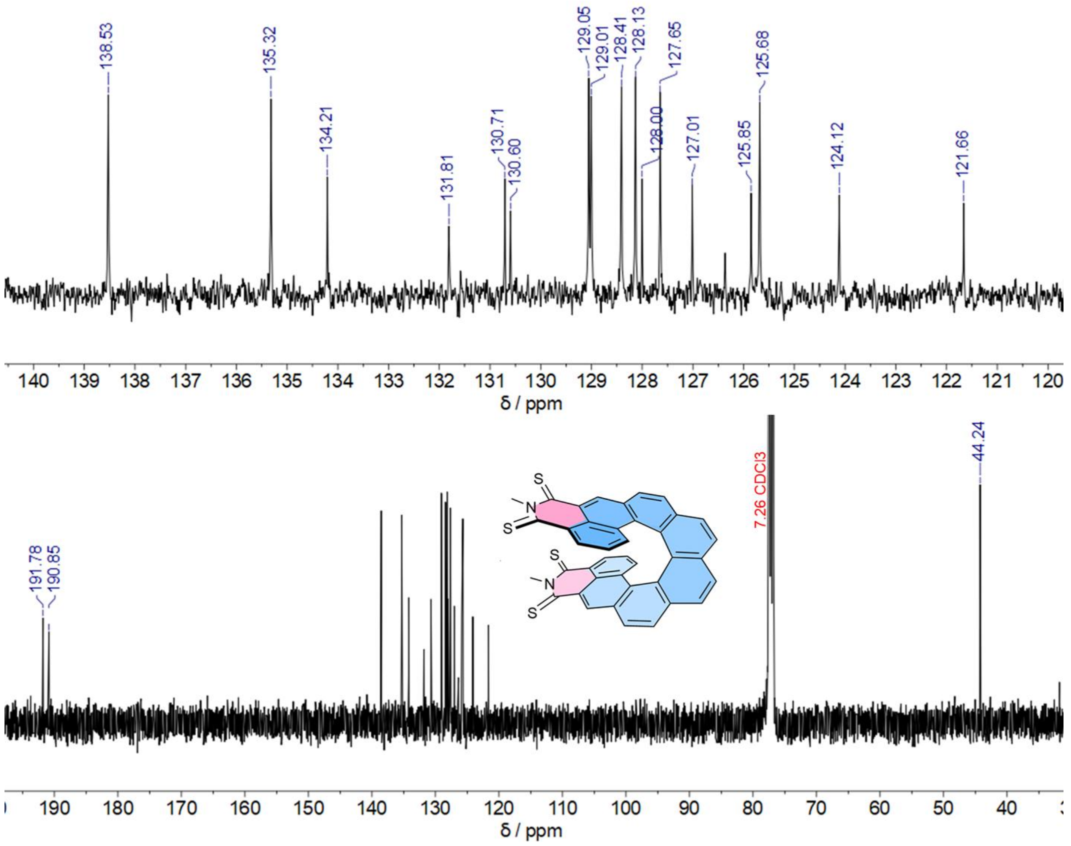

Figure S2. ^{1}H (400 MHz, $CDCl_3$) and ^{13}C (101 MHz, $CDCl_3$) NMR spectra of **[8]HDI_S4.**

S3. Spectroscopy

All compounds exhibited good solubility in common organic solvents such as dichloromethane, chloroform, THF, and aromatic solvents like toluene, but were insoluble in protic solvents (e.g., methanol) and non-polar solvents (e.g., hexane). Enantiopure samples generally displayed significantly better solubility than their racemic counterparts

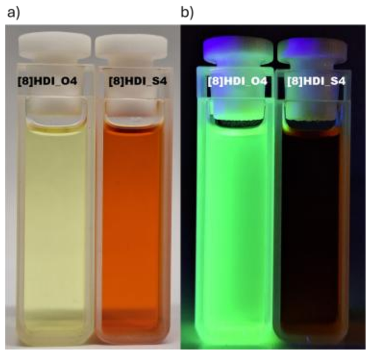

Figure S3. Photographs of **[8]HDI_O4** and **[8]HDI_S4** under (a) ambient light and (b) under 365 nm UV light. The compounds were dissolved in DCM ($c \sim 10^{-4}$ M).

We attempted to measure fluorescence and possible phosphorescence from [8]HDI_S4, including in the NIR region, however, no detectable emission was observed under the applied experimental conditions. This observation is consistent with sulfur-induced quenching, enhanced non-radiative decay, and increased spin–orbit coupling in the thionated derivative.

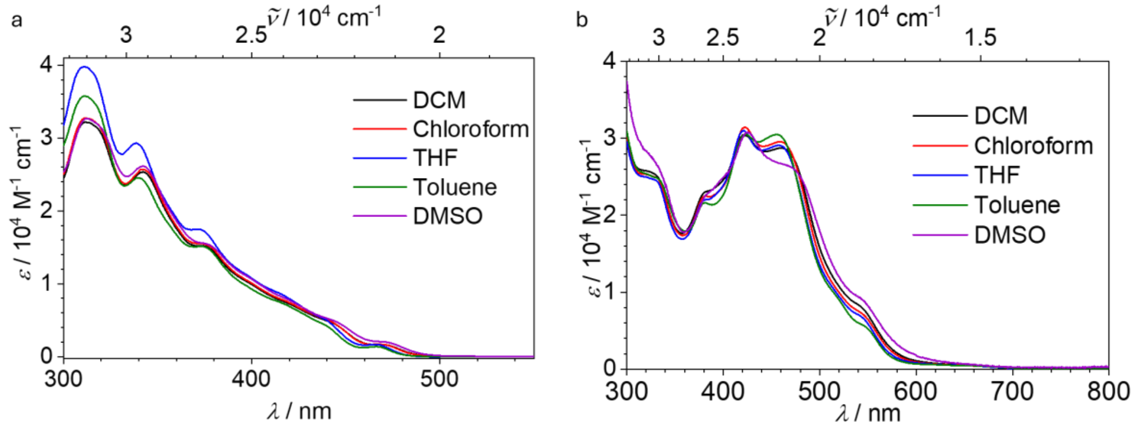

Figure S4. UV–vis absorption spectra of (a) **[8]HDI_O4** and (b) **[8]HDI_S4** (1×10^{-5} M) recorded in DCM, chloroform, THF, toluene, and DMSO.

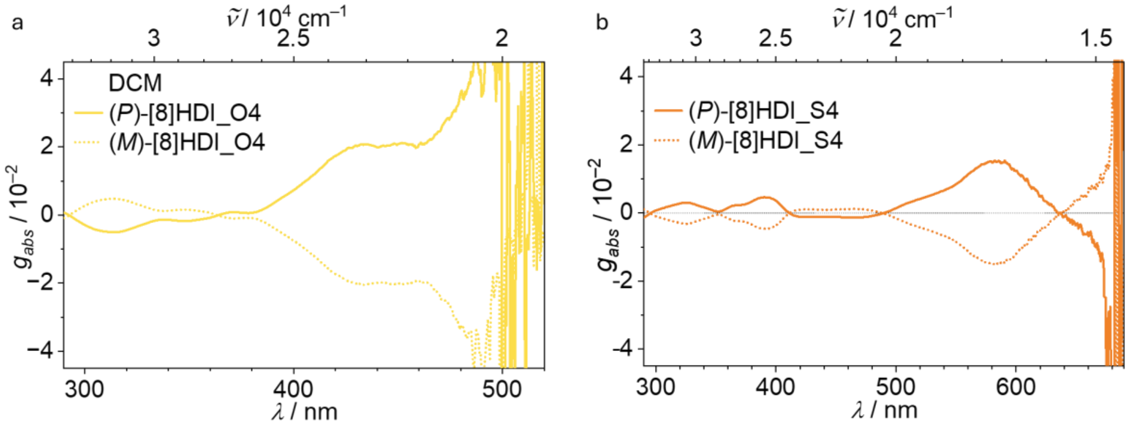

Figure S5. Absorption dissymmetry factor (g_{abs}) spectra of (a) [8]HDI_O4 and (b) [8]HDI_S4, (~1×10^{-5} M) recorded in DCM.

S4. Electrochemistry

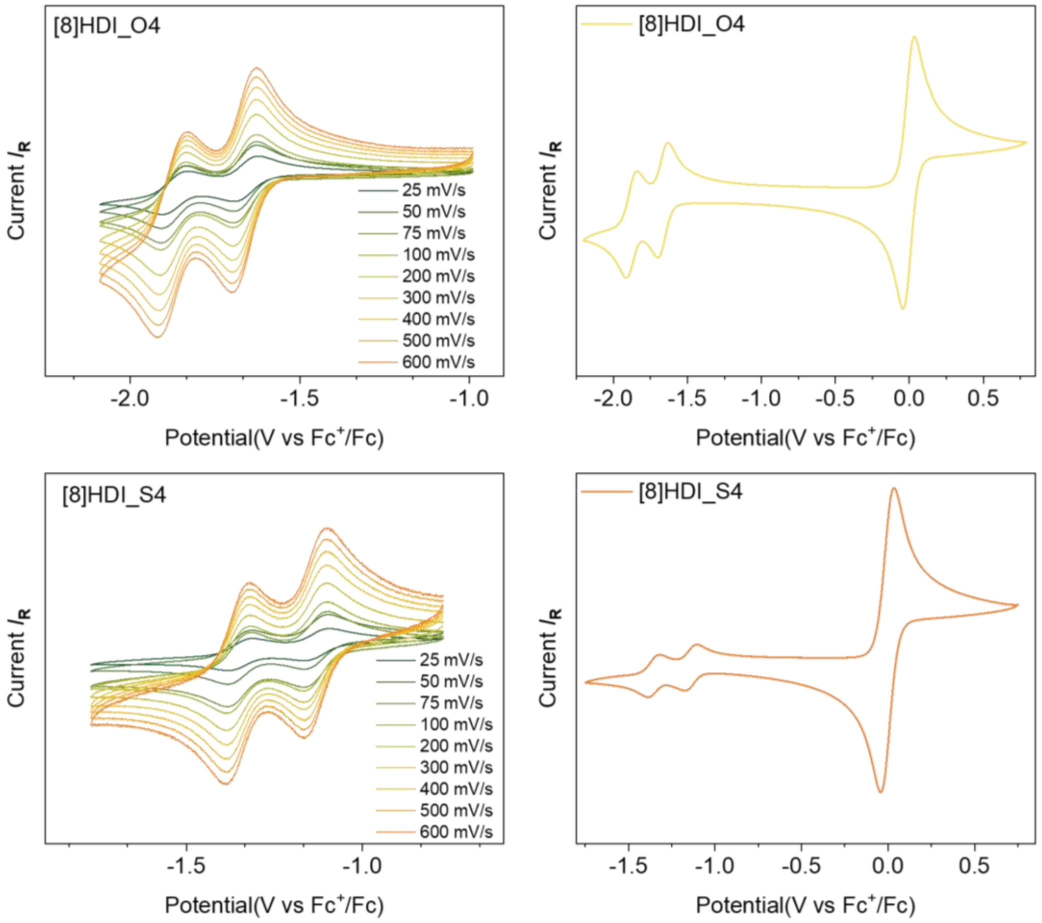

Figure S6. CV spectra of the **[8]HDI_O4** and **[8]HDI_S4** in DCM supported by a 0.2 M solution of $[Bu_4N][PF_6]$ as supporting electrolyte at various scan speeds (left) and the full CV spectrum including the Fc/Fc^+ reference at 200 mV/s (right).

Table S1. Summary of electrochemical parameters in DCM.

	$E_{red}1$ / V	$E_{red}2$ / V	ΔE_{red} [a] / V	LUMO[b] / eV	HOMO[c]/ eV	K_c[d]
**[8]HDI_O4**	−1.65	−1.87	0.22	−3.45	−5.98	5.1×10^3
**[8]HDI_S4**	−1.13	−1.35	0.22	−3.97	−5.74	5.1×10^3

[a]Potential difference between two reduction waves vs Fc/Fc+ in DCM, calculated from the peak of the DPV curves. [b]LUMO = $-(5.1 + E_{red}1)$ eV.[4] [c] HOMO = LUMO – $E_{g(opt)}$, [d]Comproportionation constant, $K_c = \exp(\Delta E_{red}F/RT)$, where F/RT = 38.92 V^{-1} at 298 K.

S5. Spectroelectrochemistry

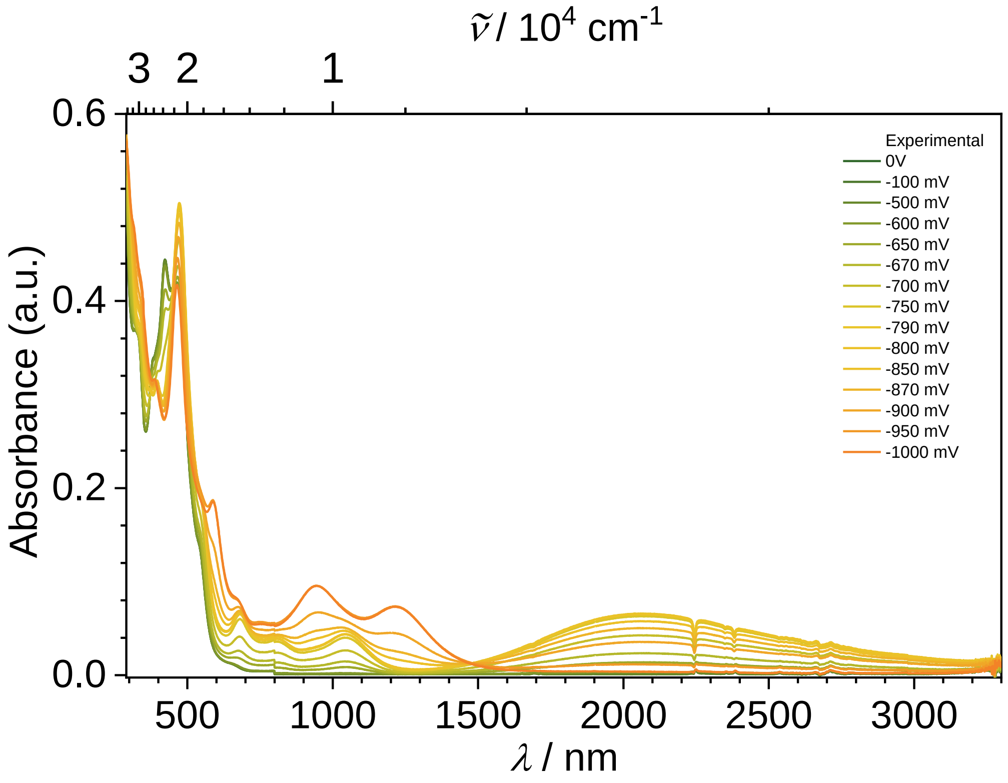

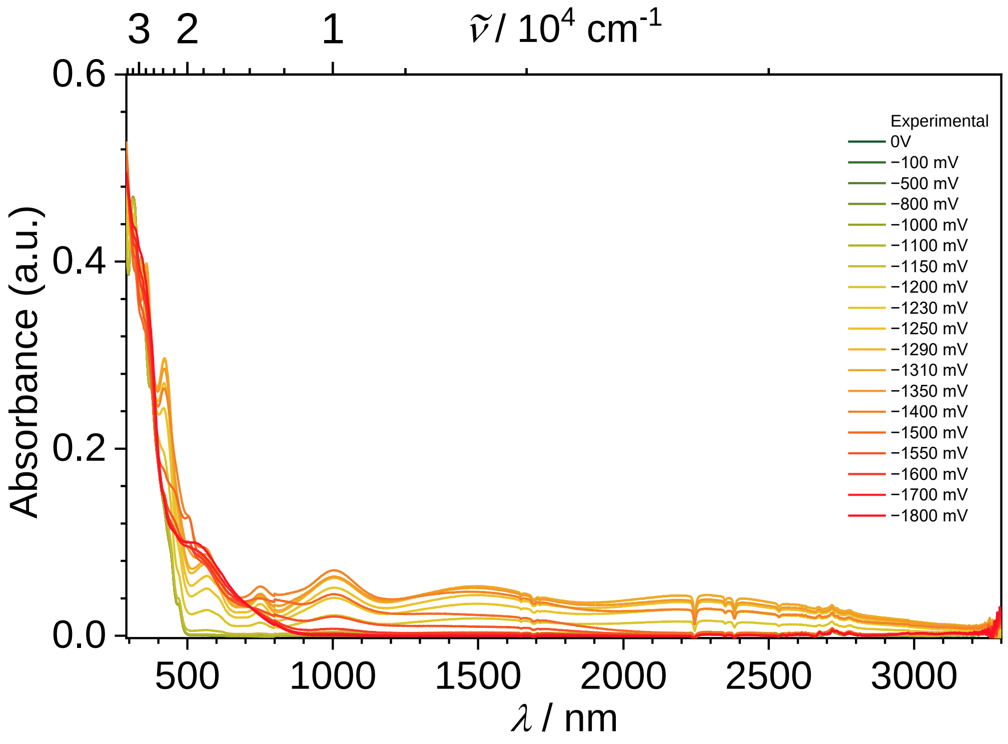

Figure S7. Spectroelectrochemical data showing the evolution of the absorption spectra during electrochemical reduction for **[8]HDI_S4** (top) and **[8]HDI_O4** (bottom).

Hush Analysis of the Intramolecular Intervalence Charge-Transfer (IV–CT) Band

The electronic coupling (V_{12}) was calculated according to Hush theory using the expression below.[5]

$$V_{12} = \frac{\sqrt{\varepsilon_{max}\ \Delta\nu_{\frac{1}{2}max}\ \nu_{max}}}{r}$$

where ν_{max} is the energy of the IV–CT band maximum (cm^{-1}), ε_{max} is the molar absorptivity ($M^{-1}\ cm^{-1}$), $\Delta\nu_{1}/_{2}$ is the full width at half maximum (cm^{-1}), and r is the distance between redox centers distance (Å).

Table S2. IV–CT band parameters and calculated electronic coupling values (V_{12}) for **[8]HDI_O4** and **[8]HDI_S4** obtained from spectroelectrochemical measurements.

	**[8]HDI_O4**	**[8]HDI_S4**
ε_{max} ($M^{-1}\cdot cm^{-1}$)	1254.78	4493.20
$\Delta\nu_{1/2}$ (FWHM) (cm^{-1})	8789.74	11555.83
r (Å)	3.406	3.472
ν_{max} (cm^{-1})	4493.22	4667.81
Hush numerator	4.956×10^{10}	2.425×10^{11}
sqrt(Hush numerator)	2.226×10^{5}	4.923×10^{5}
V_{12} (cm^{-1})	1346.10	2920.94

S6. Transient Absorption

Femtosecond Transient Absorption (fs-TA)

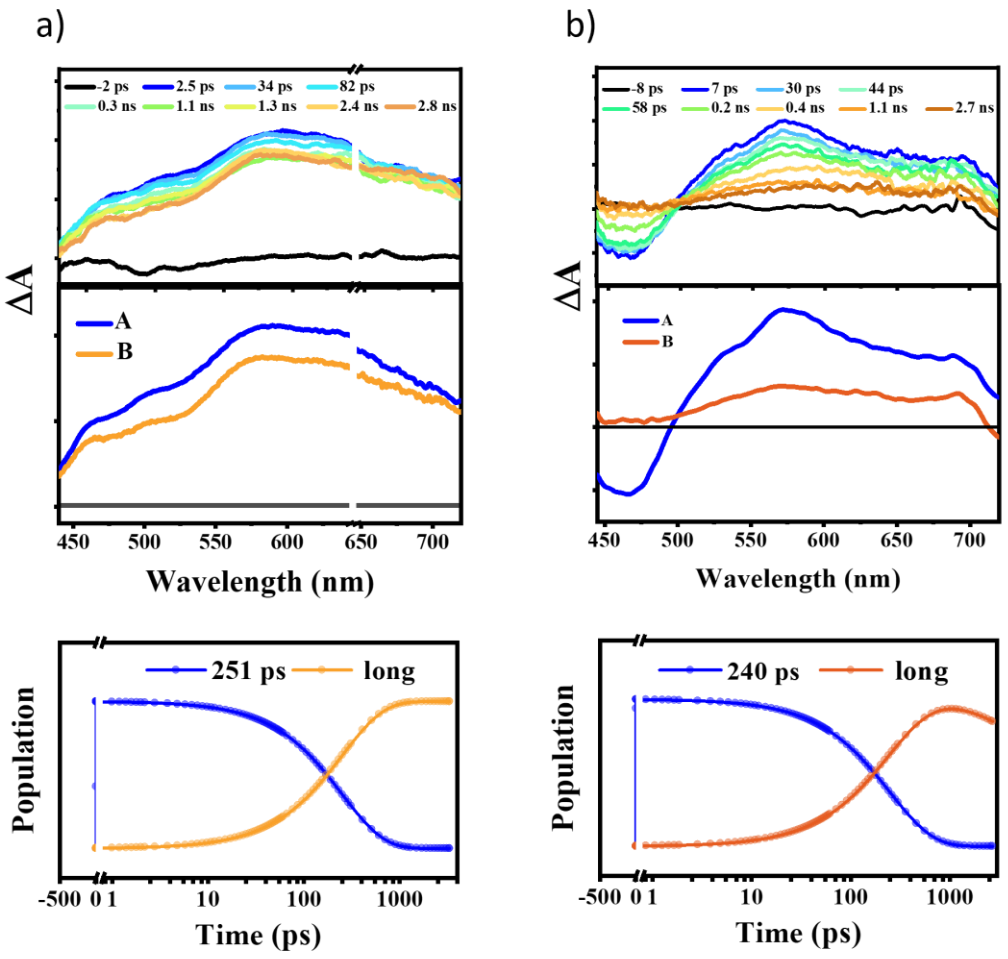

Figure S8. Femtosecond transient absorption spectra of (a) **[8]HDI_O4** and (b) **[8]HDI_S4** recorded in chloroform upon excitation at λ_{ex} = 400 nm. Top panels show the time-resolved transient absorption spectra. Middle panels display the species-associated difference spectra obtained from global target analysis. Bottom panels present the corresponding relative population profiles of the excited states.

Nanosecond Transient Absorption (ns-TA)

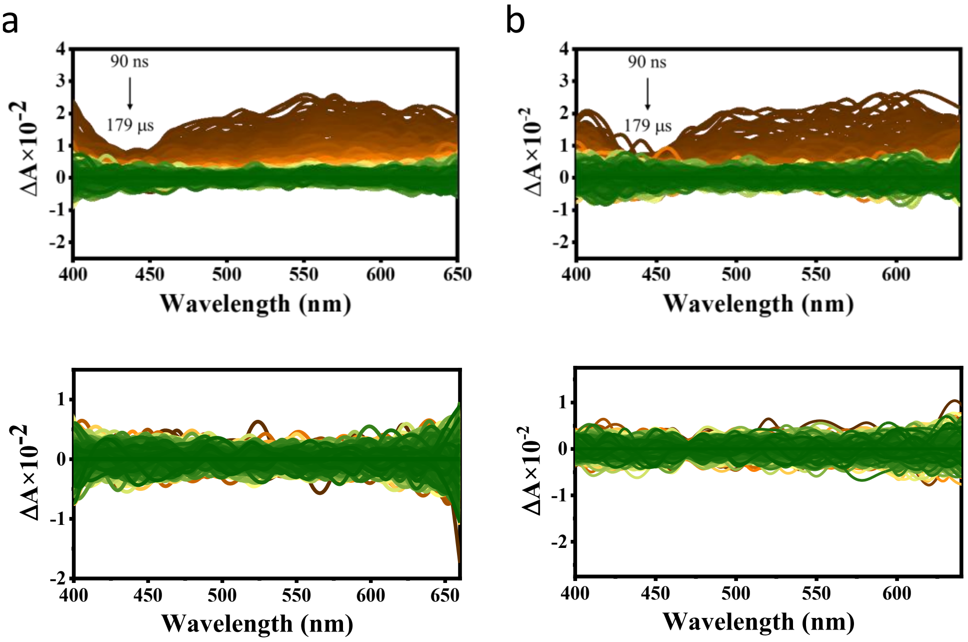

Figure S9. Nanosecond transient absorption spectra of **[8]HDI_O4** recorded in (a) chloroform and (b) toluene under N_2-purged (top panels) and O_2-purged conditions (bottom panels).

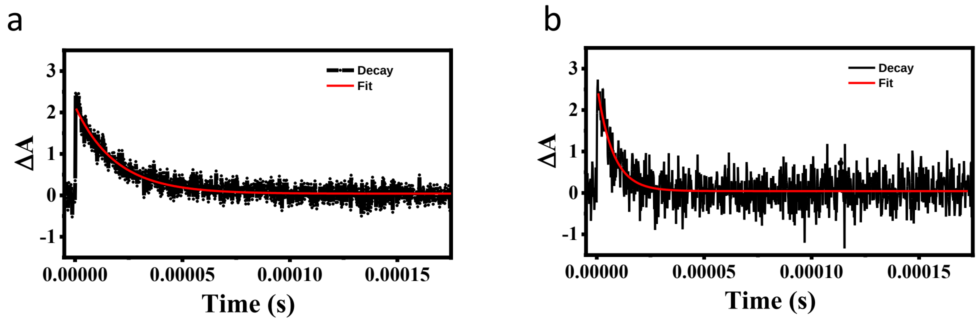

Figure S10. Single wavelength decay kinetics fitted at 600 nm yielding the triplet excited lifetime in [8]HDI_O4 recorded in (a) chloroform and (b) toluene under N_2-purged conditions.

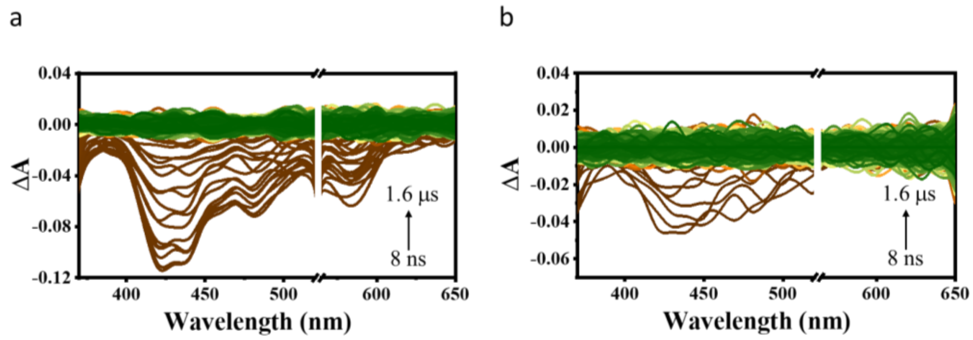

Figure S11. Nanosecond transient absorption spectra of [8]HDI_S4 recorded in (a) chloroform and (b) toluene under N_2-purged conditions.

S7. Quantum Chemical Calculations

DFT calculations were performed using Gaussian 16 suite.[6] Geometries were optimized using ωB97XD functional and 6-31g(d,p) basis set in the gas phase. TD-DFT calculations were performed on ωB97XD/6-31g(d,p) optimized geometries at the B3LYP/6-311G(2d,p) level. The effect of the solvent was accounted using PCM (with toluene as the solvent). SpecDis and Avogadro software were used to analyze the TD-DFT calculated spectra and POV-Ray was used to render graphical images of frontier molecular orbitals (FMOs). Spin–orbit coupling (SOC) matrix elements between electronic states were computed using the PySOC package.

	[8]HDI_O4	[8]HDI_S4
**LUMO+2**	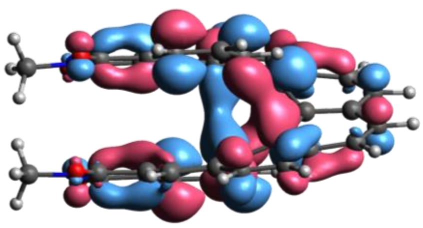	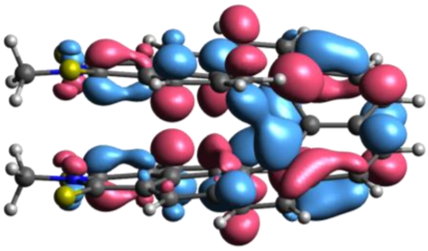
**LUMO+1**	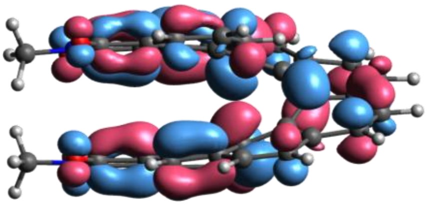	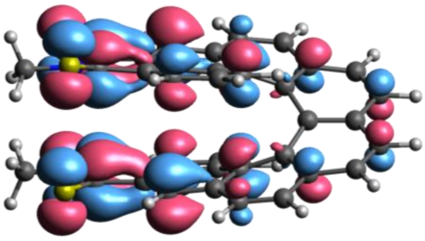
**LUMO**	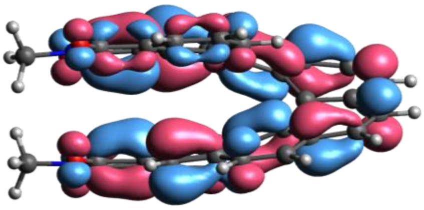	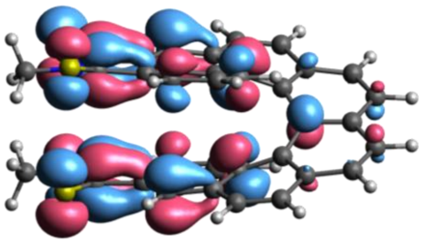
**HOMO**	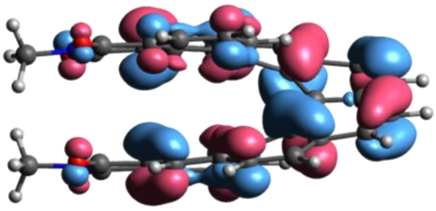	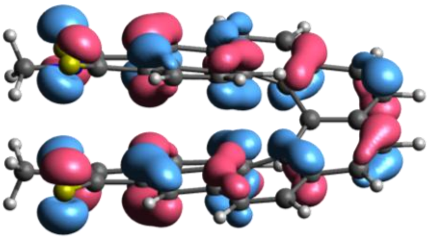
**HOMO–1**	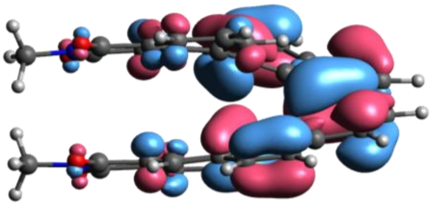	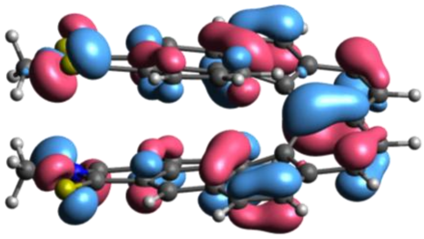
**HOMO–2**	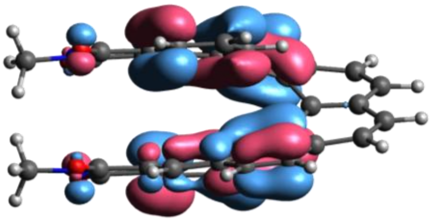	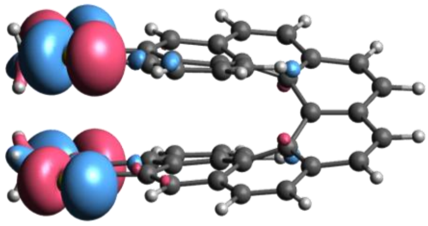

Figure S12. Frontier molecular orbitals of the discussed compounds.

Table S3. B3LYP/6-311G(2d,p) calculated parameters defining the g_{abs} for the discussed compounds in toluene.

	λ [a]	f [b]	$\|\mu\|$ [c]	$\|m\|$ [d]	$\cos\theta$ [e]	R [f]	$10^2 \times g_{abs}$ [g] (calc.)	$10^2 \times g_{abs}$ [g] (obs.)
**[8]HDI_O4**	453.9	0.0146	118.80	1.403	0.986	164.45	4.66	3.08
**[8]HDI_S4**	651.2	0.0001	10.51	0.0448	0.930	0.436	1.58	1.05

[a] Wavelength in nanometers. [b] Oscillator strength in atomic units. [c] Electric transition dipole moments in 10^{-20} esu · cm. [d] Magnetic transition dipole moments in10^{-20} esu · cm. [e] Angle between m and μ in degrees. [f] Rotational strength in erg · esu · cm / Gauss. [g] Dimensionless values.

Table S4. Summary of B3LYP/6-311G(2d,p) calculated key low-energy transitions.

**Excited singlet state**	**Wavelength**	**Energy**	**Major transitions**	**Contribution**	**Oscillator strength (*f*)**
			**[8]HDI_O4**		
**1**	453.90 nm	2.73 eV	HOMO–1 → LUMO	0.65	0.0146
			HOMO → LUMO+1	–0.22	
**2**	438.56 nm	2.82 eV	HOMO–1 → LUMO	0.23	0.0322
			HOMO → LUMO+1	0.65	
			HOMO → LUMO+2	0.13	
			**[8]HDI_S4**		
**1**	651.21 nm	1.90 eV	HOMO−2 → LUMO+1	0.10	0.0001
			HOMO−1 →LUMO	0.33	
			HOMO →LUMO+1	0.05	
**2**	650.35 nm	1.91 eV	HOMO−2→LUMO	0.22	0.0004
			HOMO−1→LUMO+1	0.15	
			HOMO→LUMO	0.10	
**3**	575.73 nm	2.15 eV	HOMO−2→LUMO	0.14	0.0180
			HOMO→LUMO	0.34	

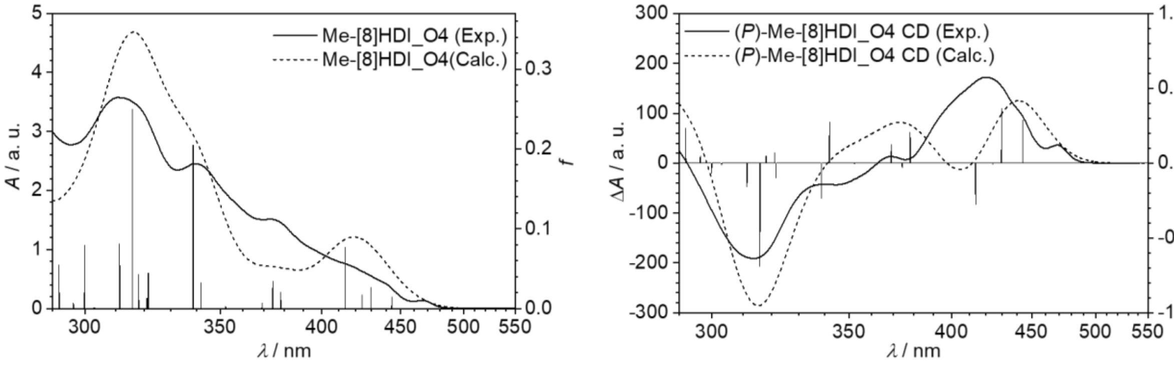

Figure S13. Comparison of experimental (solid line), TD-DFT calculated curve (dotted line), and bar (black bar) UV–vis spectra (left) and ECD spectra (right) of **[8]HDI_O4** in toluene.

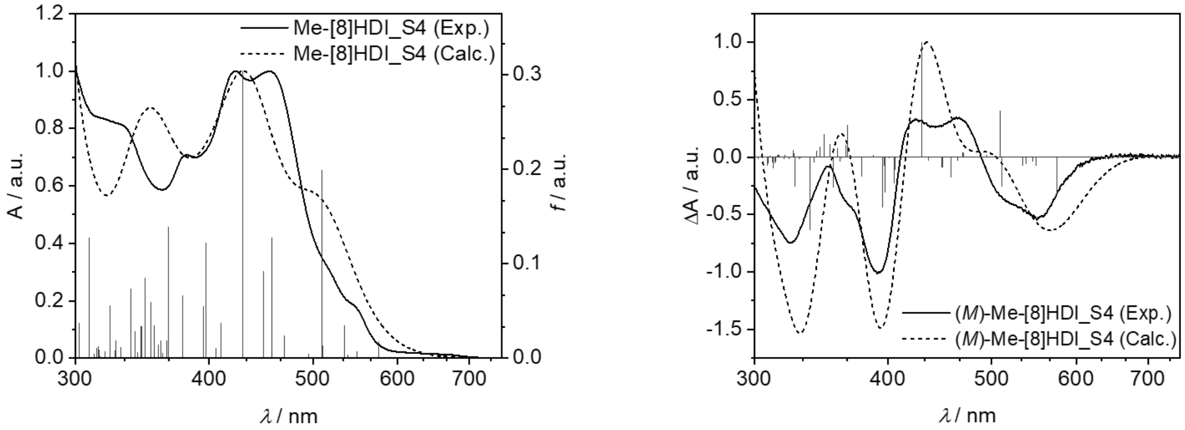

Figure S14. Comparison of experimental (solid line), TD-DFT calculated curve (dotted line), and bar (black bar) UV–vis spectra (left) and ECD spectra (right) of **[8]HDI_S4** in toluene.

Table S5. IV-CT parameters calculated for radical anions in THF using exact 35% HF-exchange contribution.[7]

	P (neutral) / Å	*P* (radical anion) / Å	E_{ab} / cm^{-1}	$\Delta\mu_{ab}$ / D	μ_{ab} / D	V_{12} / cm^{-1}	ΔG_{ab} / cm^{-1}
**[8]HDI_O4**(–1)	3.461	3.407	3503.3	10.0	2.04	661	340
**[8]HDI_S4** (–1)	3.489	3.472	3672.6	14.1	4.57	997	192

$\Delta\mu_{ab}$: Dipole moment difference between ground state and IV-CT state, μ_{ab}: projection of transition moment on dipole moment difference vector $\Delta\mu_{ab}$. H_{ab}: Electronic coupling between the redox centers.

Table S6. TD-DFT Calculated Singlet Excited States of **[8]HDI_O4**. Vertical excitation energies (eV) and oscillator strengths (f) for the first ten singlet excited states (S_1–S_{10}) computed at the B3LYP/6-31G(d,p) level of theory.

States	Energy (eV)	Oscillator Strength (*f*)
S1	2.7261	0.0162
S2	2.9100	0.0066
S3	3.0243	0.0262
S4	3.0571	0.0076
S5	3.1126	0.0528
S6	3.2320	0.0102
S7	3.3835	0.0575
S8	3.4493	0.0161
S9	3.5312	0.0012
S10	3.5574	0.0136

Table S7. TD-DFT Calculated Triplet Excited States of **[8]HDI_O4.** Vertical excitation energies (eV) of the first ten triplet excited states (T_1–T_{10}) computed at the B3LYP/6-31G(d,p) level of theory.

States	Energy (eV)
T1	2.2687
T2	2.3614
T3	2.6098
T4	2.6962
T5	2.7842
T6	2.8315
T7	3.0572
T8	3.1039
T9	3.1285
T10	3.2066

Table S8. TD-DFT Calculated Singlet Excited States of **[8]HDI_S4**. Vertical excitation energies (eV) and oscillator strengths (f) for the first ten singlet excited states (S_1–S_{10}) computed at the B3LYP/6-31G(d,p) level of theory.

States	Energy (eV)	Oscillator Strength (*f*)
S1	1.8638	0.0003
S2	1.8833	0.0006
S3	2.1935	0.0018
S4	2.2117	0.0011
S5	2.2380	0.0042
S6	2.2643	0.0095
S7	2.3425	0.0055
S8	2.3856	0.0064
S9	2.4993	0.0258
S10	2.5832	0.0656

Table S9. TD-DFT Calculated Triplet Excited States of **[8]HDI_S4.** Vertical excitation energies (eV) of the first ten triplet excited states (T_1–T_{10}) computed at the B3LYP/6-31G(d,p) level of theory.

States	Energy (eV)
T1	1.6316
T2	1.6468
T3	1.7876
T4	1.8085
T5	1.9973
T6	2.0116
T7	2.0634
T8	2.1023
T9	2.1940
T10	2.2081

Table S10. Spin-orbit coupling values between the singlet and triplet states of optimized geometry of [8]HDI_O4 computed at ωB97XD/6-31g(d,p) level of theory.

Transition	cm^{-1}	Transition	cm^{-1}
<S0|Hso|T1	0.3223	<S1|Hso|T1	1.2875
<S0|Hso|T2	0.0794	<S1|Hso|T2	0.2819
<S0|Hso|T3	0.2511	<S1|Hso|T3	0.6880
<S0|Hso|T4	0.8300	<S1|Hso|T4	0.1769
<S0|Hso|T5	1.5538	<S1|Hso|T5	0.3392
<S0|Hso|T6	2.0381	<S1|Hso|T6	0.1088
<S0|Hso|T7	0.6230	<S1|Hso|T7	0.1528
<S0|Hso|T8	0.8945	<S1|Hso|T8	0.0792
<S0|Hso|T9	2.6627	<S1|Hso|T9	0.2378
<S0|Hso|T10	0.6157	<S1|Hso|T10	0.2750

Table S11. Spin-orbit coupling values between the singlet and triplet states of optimized geometry of [8]HDI_S4 computed at ωB97XD/6-31g(d,p) level of theory.

Transition	cm $^{-1}$	Transition	cm^{-1}
<S0|Hso|T1	9.8835	<S1|Hso|T1	50.1890
<S0|Hso|T2	16.2846	<S1|Hso|T2	47.4767
<S0|Hso|T3	35.4258	<S1|Hso|T3	26.2191
<S0|Hso|T4	145.3850	<S1|Hso|T4	48.5317
<S0|Hso|T5	81.9186	<S1|Hso|T5	58.7814
<S0|Hso|T6	95.4284	<S1|Hso|T6	17.3573
<S0|Hso|T7	94.4172	<S1|Hso|T7	17.4181
<S0|Hso|T8	17.6210	<S1|Hso|T8	18.2680
<S0|Hso|T9	46.0931	<S1|Hso|T9	38.0311
<S0|Hso|T10	13.7143	<S1|Hso|T10	11.3262

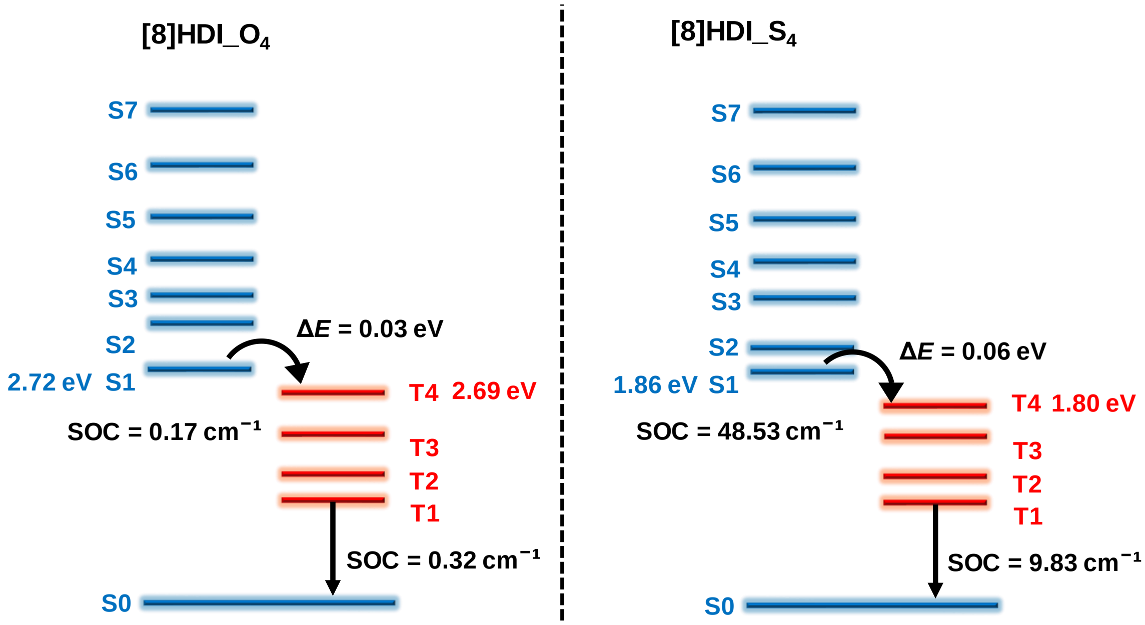

Figure S15. Excited-state energy diagrams of **[8]HDI_O4** (left) and **[8]HDI_S4** (right) illustrating the role of spin–orbit coupling (SOC) in intersystem crossing (ISC). While both systems exhibit comparable singlet–triplet energy alignment, **[8]HDI_S4** shows markedly enhanced SOC values for S_1–T_4 (48.53 cm^{-1}) and S_0–T_1 (9.83 cm^{-1}) compared to **[8]HDI_O4** S_1–T_4 (0.17 cm^{-1}) and S_0–T_1 (0.32 cm^{-1}) calculated using ωB97XD/6-31g(d,p) level of theory.

S8. References

1.F. Saal, V. Brancaccio, K. Radacki, H. Braunschweig and P. Ravat, *Angew. Chem. Int. Ed.*, 2025, **64**, e202508779.

2. (a) M. Strohalm, M. Hassman, B. Košata and M. Kodíček, *Rapid Commun. Mass Spectrom.*, 2008, **22**, 905; (b) M. Strohalm, D. Kavan, P. Novák, M. Volný and V. Havlíček, *Anal. Chem.*, 2010, **82**, 4648; (c) T. H. J. Niedermeyer and M. Strohalm, *PLOS ONE*, 2012, **7**, e44913.

3. (a) R. H. Martin and M. J. Marchant, *Tetrahedron*, 1974, **30**, 347; (b) J. Barroso, J. L. Cabellos, S. Pan, F. Murillo, X. Zarate, M. A. Fernandez-Herrera and G. Merino, *Chem. Commun.*, 2018, **54**, 188; (c) P. Ravat, *Chem. Eur. J.*, 2021, **27**, 3957.

4.C. M. Cardona, W. Li, A. E. Kaifer, D. Stockdale and G. C. Bazan, *Adv. Mater.*, 2011, **23**, 2367.

5. (a) N. S. Hush, *Progress in Inorganic Chemistry*, 1967, 391; (b) D. M. D'Alessandro and F. R. Keene, *Chemical Society Reviews*, 2006, **35**, 424.

6.M. J. Frisch, G. W. Trucks, H. B. Schlegel, G. E. Scuseria, M. A. Robb, J. R. Cheeseman, G. Scalmani, V. Barone, G. A. Petersson, H. Nakatsuji, X. Li, M. Caricato, A. V. Marenich, J. Bloino, B. G. Janesko, R. Gomperts, B. Mennucci, H. P. Hratchian, J. V. Ortiz, A. F. Izmaylov, J. L. Sonnenberg, Williams, F. Ding, F. Lipparini, F. Egidi, J. Goings, B. Peng, A. Petrone, T. Henderson, D. Ranasinghe, V. G. Zakrzewski, J. Gao, N. Rega, G. Zheng, W. Liang, M. Hada, M. Ehara, K. Toyota, R. Fukuda, J. Hasegawa, M. Ishida, T. Nakajima, Y. Honda, O. Kitao, H. Nakai, T. Vreven, K. Throssell, J. A. Montgomery Jr., J. E. Peralta, F. Ogliaro, M. J. Bearpark, J. J. Heyd, E. N. Brothers, K. N. Kudin, V. N. Staroverov, T. A. Keith, R. Kobayashi, J. Normand, K. Raghavachari, A. P. Rendell, J. C. Burant, S. S. Iyengar, J. Tomasi, M. Cossi, J. M. Millam, M. Klene, C. Adamo, R. Cammi, J. W. Ochterski, R. L. Martin, K. Morokuma, O. Farkas, J. B. Foresman and D. J. Fox, Gaussian 16 Rev. C.01; Wallingford, CT, 2016.

7. (a) C. Lambert and G. Nöll, *J. Am. Chem. Soc.*, 1999, **121**, 8434; (b) M. Renz, K. Theilacker, C. Lambert and M. Kaupp, *J. Am. Chem. Soc.*, 2009, **131**, 16292.
